# Regulation of myofibroblast dedifferentiation in pulmonary fibrosis

**DOI:** 10.1186/s12931-024-02898-9

**Published:** 2024-07-18

**Authors:** Xuetao Ju, Kai Wang, Congjian Wang, Chenxi Zeng, Yi Wang, Jun Yu

**Affiliations:** 1 Department of Thoracic Surgery, Tongji Hospital, Tongji Medical College, Huazhong University of Science and Technology, Wuhan, 430030 Hubei Province People’s Republic of China; 2 Department of Pulmonary and Critical Care Medicine, NHC Key Laboratory of Respiratory Diseases, Tongji Hospital, Tongji Medical College, Huazhong University of Science and Technology, Wuhan, 430030 Hubei Province People’s Republic of China

**Keywords:** Fibroblast, Myofibroblast, Dedifferentiation, Pulmonary fibrosis, TGF-β signaling

## Abstract

Idiopathic pulmonary fibrosis is a lethal, progressive, and irreversible condition that has become a significant focus of medical research due to its increasing incidence. This rising trend presents substantial challenges for patients, healthcare providers, and researchers. Despite the escalating burden of pulmonary fibrosis, the available therapeutic options remain limited. Currently, the United States Food and Drug Administration has approved two drugs for the treatment of pulmonary fibrosis—nintedanib and pirfenidone. However, their therapeutic effectiveness is limited, and they cannot reverse the fibrosis process. Additionally, these drugs are associated with significant side effects. Myofibroblasts play a central role in the pathophysiology of pulmonary fibrosis, significantly contributing to its progression. Consequently, strategies aimed at inhibiting myofibroblast differentiation or promoting their dedifferentiation hold promise as effective treatments. This review examines the regulation of myofibroblast dedifferentiation, exploring various signaling pathways, regulatory targets, and potential pharmaceutical interventions that could provide new directions for therapeutic development.

## Background

Currently, the estimated prevalence rate of pulmonary fibrosis per 10,000 individuals ranges from 0.57 to 4.51 in the Asia-Pacific region, 0.33 to 2.51 in Europe, and 2.40 to 2.98 in North America [1]. This condition imposes significant suffering on patients and places a heavy burden on healthcare systems and financial resources. Despite these challenges, treatment options remain severely limited. To date, only two drugs, nintedanib and pirfenidone, have been approved by the United States Food and Drug Administration for the treatment of pulmonary fibrosis. However, their effectiveness is limited; they cannot reverse fibrotic progression and are associated with significant side effects. [2–4]. Non-pharmacological interventions, such as palliative care, pulmonary rehabilitation, lung transplantation, and the management of complications and acute exacerbations, aim to alleviate symptoms and enhance quality of life but do not reverse pulmonary fibrosis.

There is an urgent need to explore and implement more treatment modalities in clinical practice. The pathogenesis of pulmonary fibrosis primarily involves sustained or repeated pulmonary epithelial injury, subsequent activation of fibroblasts, and differentiation into myofibroblasts. This process results in excessive extracellular matrix (ECM) deposition, distortion of normal lung architecture, and irreversible loss of lung function [5]. Myofibroblast differentiation plays a crucial role in the pathological physiology of pulmonary fibrosis. Although traditionally believed to be terminally differentiated cells, recent research consistently demonstrates the dedifferentiation capacity of myofibroblasts, suggesting that dedifferentiation may contribute to the regression of established fibrosis.

## Myofibroblast

### What is a myofibroblast

Myofibroblast are a type of fibroblast-like cells that contain myosin and actin, along with other muscle proteins [6]. Additionally, myofibroblasts function as intermediate cells positioned between fibroblasts and smooth muscle cells. Extensively present at wound in healing sites and fibrotic organs with high remodeling capacities, such as the lungs, kidneys, liver, and skin, myofibroblasts are the primary effector cells in the fibrotic processes of various organs [7]. They are capable of producing a substantial amount of extracellular matrix (ECM) proteins, including type I collagen and fibronectin, thereby facilitating wound healing and the organ fibrotic response [8]. Currently, smooth muscle actin (SMA) is the most commonly used biomarker to distinguish myofibroblasts from fibroblasts and other precursors. In addition, vimentin, fibroblast-specific protein 1 (FSP-1) and cadherin-11 are potential markers for fibroblast cells. Angiotensin 1 receptor (AT 1), transforming growth factor-β type II receptor (TβRII), paxillin, tensin, fibronectin extra dominant A splice variant, Frizzled-2, osteopontin, Tenascin C and periostin are highly expressed or exclusively expressed in myofibroblasts, may serve as potential markers for myofibroblasts [9].

### Origin of myofibroblasts

**Fig. 1:**
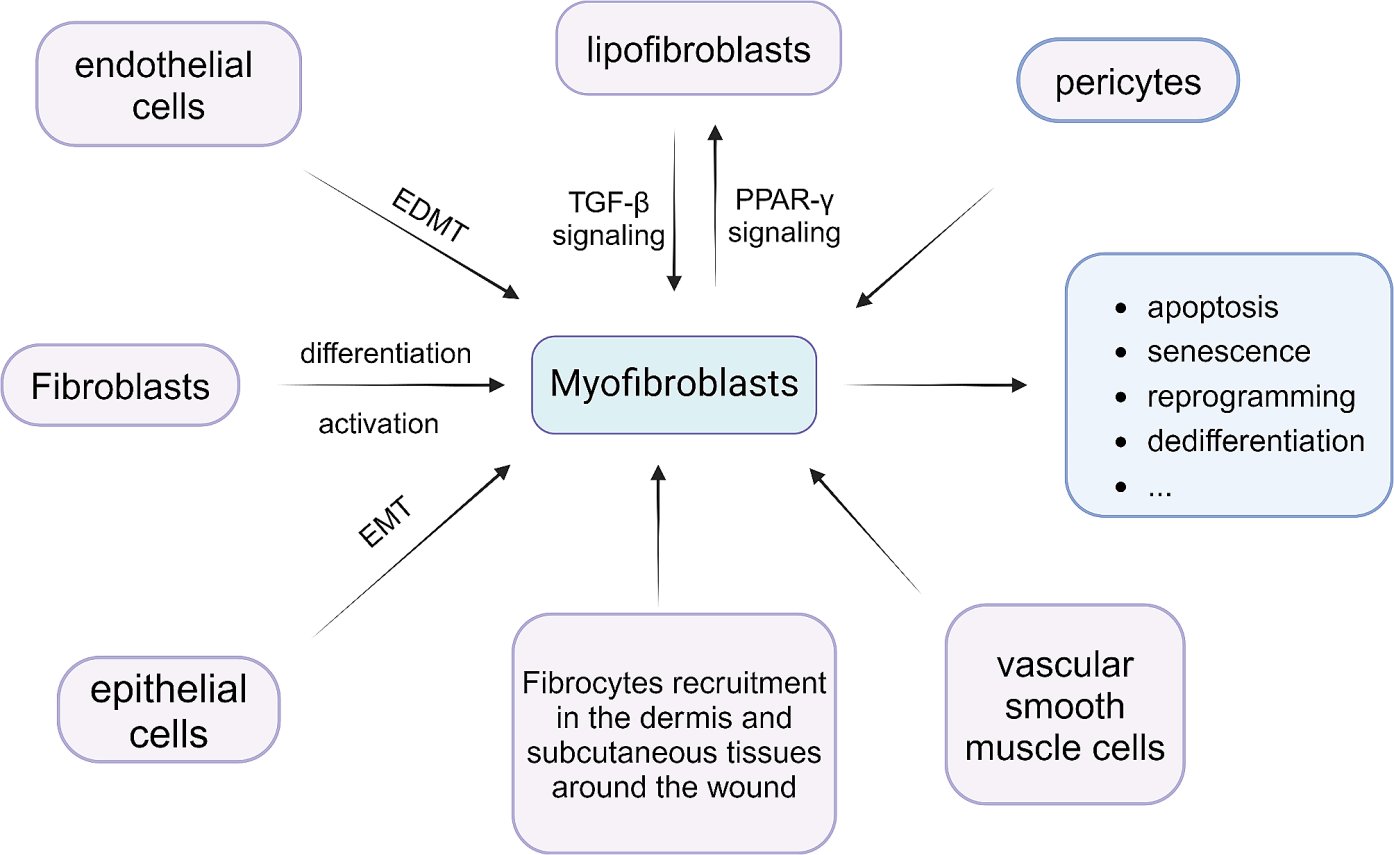
Several common origins and eliminating ways of myofibroblasts. EMT, epithelial-mesenchymal transition. EDMT, endothelial-mesenchymal transition. TGF-β, Transforming growth factor-β. PPAR-γ, peroxisome proliferator-activated receptor-γ

Myofibroblasts have several common origins. Firstly, fibroblasts can differentiate into myofibroblasts induced by the TGF-β/SMAD pathway or platelet-derived growth factor (PDGF) [10]. Secondly, there are epithelial-mesenchymal transition (EMT) and endothelial-mesenchymal transition (EDMT) [11]. Other sources include the recruitment of fibrocytes from the dermis and subcutaneous tissue around wounds, the transformation of perivascular cells and vascular smooth muscle cells into myofibroblasts [12], and the interconversion between lipogenic and myogenic fibroblastic phenotypes [13]. The origins and pathways of elimination of myofibroblasts are depicted in Fig. 1.

### Myofibroblast dedifferentiation and its therapeutic implications

The treatment of pulmonary fibrosis can be approached through several strategies: eliminating the underlying causes of fibrosis, degrading and removing fibrotic extracellular matrix (ECM), inhibiting the proliferation of fibroblasts and their differentiation into myofibroblasts, and eliminating myofibroblasts, which involves inducing apoptosis, senescence, reprogramming, dedifferentiation, and other processes [7]. Among these, dedifferentiation refers to a process wherein mature cells, which have already undergone differentiation or partial differentiation, regress from their mature state to adopt a less differentiated cellular phenotype. For instance, endothelial cells undergoing dedifferentiation transform into endothelial progenitor cells. Myofibroblast dedifferentiation refers to a significant reduction in ECM production, a decrease in the expression of α-smooth muscle actin (α-SMA) in myofibroblasts, and a lower binding affinity with stress fibers, possibly even restoring the inactive phenotypic characteristics of myofibroblast precursor cells [14, 15]. Because myofibroblast dedifferentiation is a relatively small and new research direction, there is no unified consensus on research methods in previous studies, so the experimental procedures from different articles are different. A frequently utilized experimental procedure involves subjecting fibroblasts to serum-starvation in DMEM for 24 h, followed by TGF-β pretreatment for 24–48 h to induce fibroblast differentiation into myofibroblasts. Subsequently, dedifferentiation factors are applied for a specific duration, culminating in the assessment of molecular expression levels of α-SMA and collagen at protein or RNA levels [16–18]. Generally, a significant decrease in α-SMA and collagen expression is regarded as indicative of dedifferentiation.

Other treatment modalities typically can only restrain the progression of pulmonary fibrosis, alleviate symptoms, and improve the quality of life. By the time fibrosis is diagnosed, tissue damage has already advanced. Therefore, inhibiting the formation of myofibroblasts and impeding fibrotic progression may not achieve a curative effect. In contrast, inducing myofibroblast dedifferentiation is believed to have the potential to reverse established pulmonary fibrosis, thereby achieving a curative outcome. Consequently, an increasing number of studies are focusing on the direction of myofibroblast dedifferentiation.

## TGF-β signaling

### TGF-β signaling in fibroblast differentiation

Transforming growth factor-β (TGF-β) serves as the prototype within the TGF-β family, which includs factors such as activin, nodal, bone morphogenetic proteins (BMPs), and growth and differentiation factors (GDFs) [19, 20]. In the differentiation process from fibroblasts to myofibroblasts, TGF-β is recognized by TGF-β receptor II (TβRII) which contains the intracellular kinase domain. This domain recruits and phosphorylates TGF-β receptor I (TβRI) through a glycine/serine-rich “GS sequence” undergoing threonine/serine kinase conversion. Subsequently, TβRII and TβRI form a heteromeric complex [21]. Activated TβRI phosphorylates R-Smad (Smad2 and Smad3) proteins, facilitating the formation of a trimeric complex with Co-Smad namely Smad4. This trimeric complex translocates into the cell nucleus, acting as a transcription factor to regulate the expression of fibrosis-related target genes, including fibronectin, collagen, plasminogen activator inhibitor-1 (PAI-1), and connective tissue growth factor (CTGF) [22–24]. This process is modulated by coactivators such as p300, CBP, activating protein-1 (AP-1), and specificity protein 1 (Sp1), or corepressors such as c-Ski, SnoN, transforming growth inhibiting factor, and Smad nuclear-interacting protein-1 [25]. Activation of the Smad signaling pathway induces the expression of transcription factors such as SNAIL, SLUG, ZEB, and TWIST, which act as inhibitors of E-cadherin and mediate desmosome dissociation, contributing to the epithelial-mesenchymal transition (EMT) [26]. Additionally, Smad pathway activation leads to Protein kinase B (Akt) activation and subsequent nuclear translocation of β-catenin, resulting in upregulation of α-SMA [27]. Negative regulators of the Smad pathway, Smad6 and Smad7, counteract TGF-β signaling by binding to type I receptors and competing with activated R-Smads for binding to Co-Smad4. Furthermore, inhibitory Smads recruit E3 ubiquitin-protein ligases Smurf1 and Smurf2, targeting Smad proteins for proteasomal degradation and thereby terminating Smad-mediated signal transduction. Smad6 expression is induced by Smad1 and 5, while Smad7 expression is triggered by Smad3 [28, 29]. IL-7 and IFN-γ induce Smad7 expression through the JAK-STAT pathway, inhibiting TGF-β signal transduction and mitigating bleomycin-induced pulmonary fibrosis [30, 31].

TGF-β can also activate the MAPK family, including extracellular signal-regulated kinase (ERK), p38 mitogen-activated protein kinase (MAPK), and Jun N-terminal kinase (JNK) signals [32]. Additionally, it can activate the phosphoinositide 3-kinase (PI3K)/Akt and Rho GTPase pathways and synergistically interact with the Wnt and Notch signaling cascades [23]. These non-Smad transducers activated by receptors can function as independent pathways or cooperatively mediate signal responses in conjunction with Smads, converging onto Smads to control their activity [33, 34]. For instance, Erk1/2 can phosphorylate transcription factors such as Fos-related antigen 2 (Fra-2), promoting gene transcription [35, 36]. Protein kinase B (Akt) can be activated by TGF-β signaling through phosphoinositide 3-kinase (PI3K), regulating translation reactions via mTOR [33]. Akt can also be activated through TRAF6-mediated Akt lysine-63 chain ubiquitination or miR-216a/217 microRNA cluster-mediated suppression of Smad7 phosphatase and tensin homolog (PTEN) in non-canonical pathways [37, 38].

**Fig. 2:**
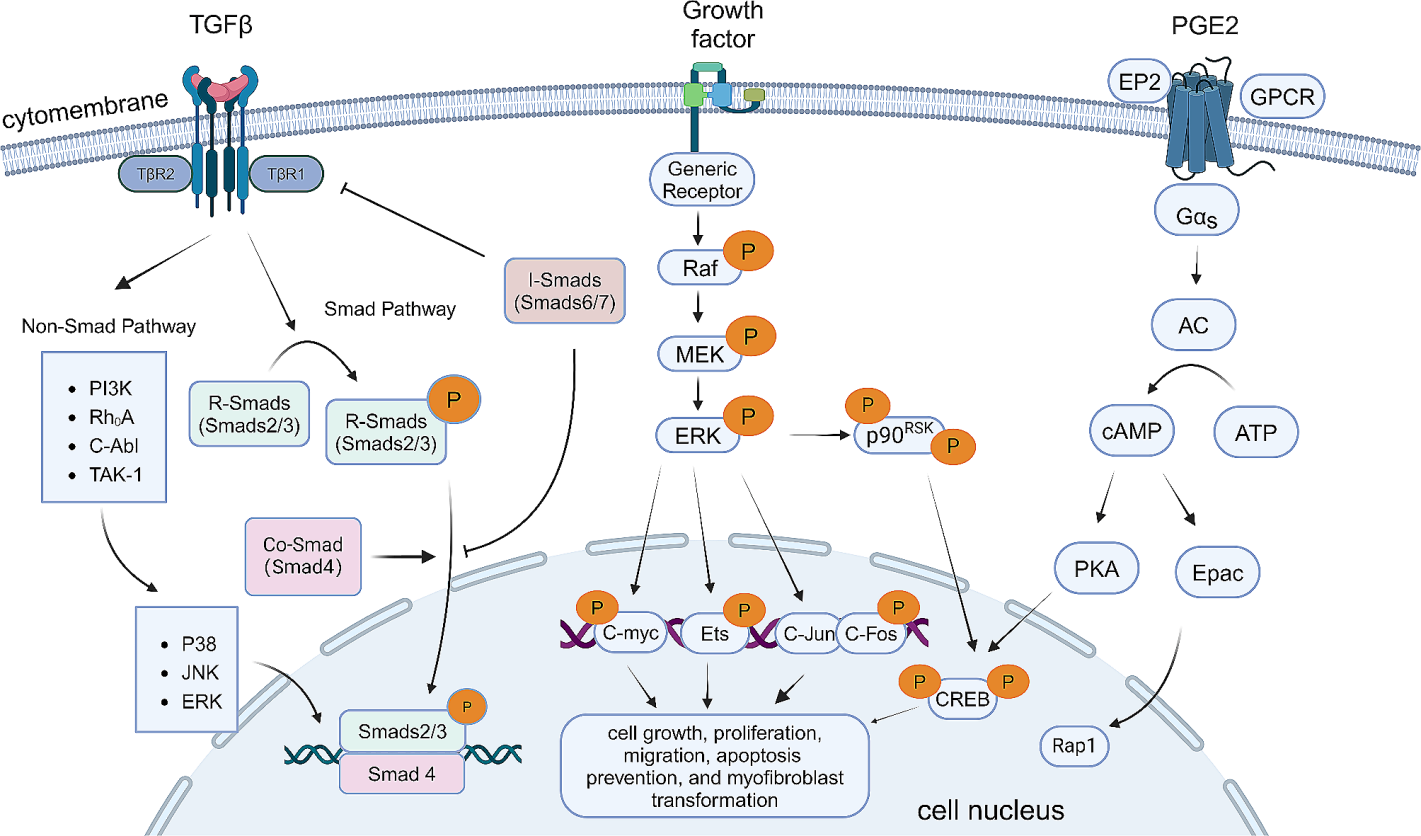
Signaling pathways involved in myofibroblast dedifferentiation, including TGF-β signaling, MEK/ERK pathway and cAMP/PKA pathway. TβR, TGF-β receptor. MEK, Mitogen-activated protein kinase kinase. ERK, Extracellular-signal-regulated kinases. GPCR, G-protein-coupled receptors. AC, adenylyl cyclase. PKA, protein kinase A. Epac, exchange protein activated by cAMP. CREB, cAMP-response element binding protein

TGF-β signaling and another two signaling pathways involved in myofibroblast dedifferentiation are demonstrated in Fig. 2.

### TGF-β signaling in myofibroblast dedifferentiation

The TGF-β signaling pathway plays a pivotal role in regulating the differentiation of fibroblasts to myofibroblasts and is equally crucial in the regulation of myofibroblast dedifferentiation. Numerous studies on treatment for idiopathic pulmonary fibrosis (IPF) focus on inhibiting the TGF-β pathway, including antagonizing TGF-β, inhibiting Smad2/3 phosphorylation, and activating Smad7 [39]. Research on myofibroblast dedifferentiation also predominantly centers on the regulation of the TGF-β pathway. Nuclear factor erythroid 2-related factor 2 (Nrf2) activators such as arsenic trioxide (ATO) and sulforaphane (SFN) inhibit Smad2/3 phosphorylation, reduce TGF-β-induced reactive oxygen species accumulation (ROS), and restore antioxidant defenses [16, 18]. Regulation of myofibroblast dedifferentiation through Ca^2+^ modulation is linked to the TGF-β pathway. Inhibitors of store-operated calcium entry (SOCE) can suppress fibrotic marker expression by inhibiting the TGF-β1/Smad3 pathway [40]. Disrupting extracellular Ca^2+^ or using a selective KCa3.1 blocker hinders Smad2/3 nuclear translocation, inducing myofibroblast dedifferentiation [41]. Cyclosporin A and HIF-1α inhibitors (HIFi) inhibit TGF-β1-induced fibroblast-to-myofibroblast transition, reducing expression levels of α-SMA and fibronectin, and dedifferentiating myofibroblast-like cells (MyoLCs) from pulmonary fibrosis patients [42]. Bromodomain and extra terminal domain (BET) protein inhibitors such as JQ1 can reverse TGF-β-mediated NOX4/SOD2 imbalance and Nrf2 inactivation, attenuating ROS production and reversing myofibroblast differentiation, thus exhibiting therapeutic effects against fibrosis [43, 44]. Bortezomib (BTZ) can inhibit TGF-β1 and key kinases activated by TGF-β1 and FGF, significantly reducing the expression of α-SMA and Collagen in TGF-β-induced myofibroblasts, thus inducing dedifferentiation [45]. Secretome components from mesenchymal stem cells (MSCs) selectively inhibit TGF-β-induced myofibroblast differentiation through the TGF-β Smad2/3 signaling pathway, facilitating reversible dedifferentiation into a fibroblast-like cell subset [46]. Peroxisome proliferator-activated receptor (PPAR) agonists can inhibit the activation of transcription factors STAT3 and EGR1 in TGF-β transgenic mouse kidneys, block TGF signaling and its effects, convert TGF into inactive monomers, and inhibit Smad2/3 phosphorylation to achieve myofibroblast dedifferentiation [47–49].

### Nuclear factor erythroid 2-related factor 2 (Nrf2)

Nrf2, encoded by nuclear factor erythroid 2-like 2 (NFE2L2), is a crucial transcription factor in oxidative stress responses. It binds to antioxidant response elements (AREs) in the promoter regions of many cell-protective genes, safeguarding various tissues and organs from oxidative damage and inflammatory stress [50, 51]. Nrf2 regulates the expression of hundreds of genes, including those for antioxidant enzymes, immune and inflammatory response limiters, and genes that restrain tissue remodeling and fibrosis [52]. NFE2L2 can inhibit fibroblast-to-myofibroblast differentiation (FMD) in IPF [53] and promote myofibroblast dedifferentiation, contributing to the reduction of fibrosis. Silencing Nrf2 with siRNA induces oxidative stress and FMD. Activating Nrf2 with keap1 siRNA downregulates the expression of α-SMA and collagen at the RNA level, enhancing antioxidant defense and myofibroblast dedifferentiation in IPF fibroblasts [18]. Arsenic trioxide (ATO), an Nrf2 activator, increases the expression of the antioxidant gene HO-1 in fibroblasts, reducing TGF-β1-induced ROS accumulation. ATO treatment strongly induces the transcription factor NFE2L2, promoting its nuclear translocation and inhibiting Smad2/3 phosphorylation [16]. Sulforaphane (SFN), another Nrf2 activator, promotes myofibroblast dedifferentiation in pulmonary fibrosis by inducing Nrf2 expression. Additionally, SFN inhibits the detrimental pro-fibrotic effects of TGF-β in IPF, controlling fibroblasts and restoring antioxidant defenses [18]. Therefore, both ATO and SFN can concurrently inhibit fibroblast differentiation and promote myofibroblast dedifferentiation, contributing to the therapeutic effects in treating pulmonary fibrosis.

### Ca^2+^

Ca^2+^ plays a crucial role in pulmonary fibrosis induced by systemic sclerosis (SSc), with most SSc pathological pathways closely associated with Ca^2+^ signaling [40]. The increased levels of TGF-β1 in SSc are positively correlated with intracellular Ca^2+^ activity. Store-operated Ca^2+^ entry (SOCE) represents a vital Ca^2+^ signal linked to inflammation and carcinogenesis [54]. SOCE channels play a crucial role in promoting fibroblast proliferation, differentiation, and ECM protein production [55]. Inhibitors of SOCE disrupt intracellular Ca^2+^ activity, leading SSc myofibroblasts to dedifferentiate into normal fibroblasts, resulting in reduced expression of α-SMA and fibronectin [56]. 2-APB, SOCE associated inhibitor, inhibits fibrosis markers such as α-SMA, fibronectin and vimentin, by suppressing TGF-β1/SMAD3 signaling. 2-APB disrupts intracellular Ca^2+^ regulation, inducing dedifferentiation of TGF-β-induced myofibroblasts. Subcutaneous injection of 2-APB ameliorates bleomycin-induced skin and lung fibrosis [40]. Other SOCE inhibitors, such as SKF96365 and indomethacin, partially achieve the above effects [56].

KCa3.1 is a Ca^2+^-activated K^+^ channel that regulates calcium signaling and maintains membrane potential during cell activation [57]. KCa3.1 modulates cell activity by regulating the proliferation, activation, migration, and mediator release of various cells, including fibroblasts. Elevated KCa3.1 expression promotes lung fibroblast proliferation and function. KCa3.1 activity has been shown to enhance Smad phosphorylation, promoting the upregulation of α-SMA in response to TGF-β1 [58–60]. In large animal models of IPF, blocking KCa3.1 channels inhibits the pro-fibrotic effects of primary sheep lung fibroblasts and mitigates bleomycin-induced pulmonary fibrosis in the early stages [61]. In human lung fibroblasts, KCa3.1 activity promotes the functionality of IPF-derived lung fibroblasts. Blocking KCa3.1 reduces TGF-β1-dependent collagen secretion in fibroblasts [59]. Disrupting extracellular Ca^2+^ or using selective KCa3.1 inhibitors (TRAM-34, ICA-17,043) to block KCa3.1 ion channels inhibits Smad2/3 nuclear translocation, significantly reduces SMA protein expression, and impedes stress fiber formation. This induces dedifferentiation of IPF-derived human lung myofibroblasts (HLMFs) toward a quiescent fibroblast phenotype [41]. Therefore, selective KCa3.1 inhibitors may offer a novel therapeutic approach for IPF, with related drugs awaiting further development.

### Hypoxia-inducible factor (HIF)-1α

Hypoxia-inducible factor (HIF)-1α serves as a critical mediator in cellular metabolism, inflammation, and tumorigenesis under hypoxic conditions. In patients with IPF, HIF-1α is highly expressed in the lungs and can induce endoplasmic reticulum (ER) stress in alveolar epithelial cells (AECs) [62]. Additionally, HIF-1α promotes EMT in AECs [63] and participates in TGF-β1-induced FMD [42].

Cyclosporine A (CsA) is a potent immunosuppressive cyclic nonapeptide [64]. Previous studies have demonstrated its efficacy in treating interstitial pneumonia, Sjogren’s syndrome, acute exacerbation of IPF, and other diseases [65–67]. These findings prompted exploration into the inhibitory effects of CsA on pulmonary fibrosis. CsA can inhibit TGF-β1-induced fibroblast-to-myofibroblast transformation by enhancing the degradation of HIF-1α protein. Moreover, CsA and HIF-1α inhibitors (HIFi) can reduce the expression levels of α-SMA and fibronectin, inducing dedifferentiation in myofibroblast-like cells (MyoLCs) derived from pulmonary fibrosis patients [42]. However, the strong toxicity of CsA [64] requires further exploration and evaluation for its clinical use in pulmonary fibrosis treatment.

### BET protein

Bromodomain and extra terminal domain (BET) proteins (Brd2, Brd3, Brd4, and BrdT) serve as epigenetic readers by binding to acetylated lysine residues on both histone and non-histone proteins through their conserved bromodomain. They regulate gene expression by recruiting transcriptional activators or repressors [68]. BET proteins have been identified as promising therapeutic targets in cancer and pulmonary fibrosis. In pulmonary fibrosis models, following stimulation by TGF-β1, Brd4 occupies the promoters of interleukin-6 (IL-6), α-smooth muscle actin (α-SMA), and plasminogen activator inhibitor-1 (PAI-1), thereby promoting fibrosis [69]. Additionally, during the pathological progression of pulmonary fibrosis, TGF-β increases NADPH oxidase-4 (NOX4), inhibits superoxide dismutase (SOD2) expression, enhances ROS production, and promotes myofibroblast differentiation. BET protein inhibitors can reverse the TGF-β-mediated imbalance of NOX4/SOD2 and Nrf2 inactivation, attenuate ROS production, and reverse myofibroblast differentiation, thus offering a potential treatment for fibrosis [43, 44].

JQ1, a small molecule inhibitor, disrupts the binding of BET proteins’ bromodomains to acetylated lysine [68]. JQ1 affects the expression of NOX4/SOD2 and Nrf2 activity to restore redox balance, leading to myofibroblast reprogramming or dedifferentiation and exerting anti-fibrotic effects [43]. Research by Kenichi Suzuki and colleagues has provided evidence supporting this notion. They demonstrated that JQ1 significantly reduces the expression levels of α-SMA and ED-A-fibronectin in primary myofibroblasts from severely fibrotic lungs and conducted the first comprehensive analysis of the transcriptome spectrum associated with the dedifferentiation of IPF myofibroblasts. Unfortunately, the exact mechanism of BET inhibition-induced myofibroblast dedifferentiation and the associated gene expression changes remain unclear, and the transcriptional profile related to IPF myofibroblast dedifferentiation remains unknown [70].

### MSC extracellular vesicles

Mesenchymal stromal/stem cells (MSCs) are adult stem cells with self-renewal capacity and multipotent differentiation potential. They exhibit robust immunoregulatory, anti-inflammatory, and anti-fibrotic properties. Research has shown that extracellular vesicles (EVs), including exosomes, derived from human bone marrow MSCs can prevent and ameliorate bleomycin-induced pulmonary fibrosis. These EVs achieve systemic regulation of monocyte phenotype, leading to improvements in lung morphology, reduced collagen deposition, and restoration of lung structure [71]. In another study, a non-contact transwell co-culture of MSCs with TGF-β-induced myofibroblasts revealed selective inhibition of the TGF-β-SMAD2/3 signaling pathway in myofibroblasts. This inhibition resulted in their reversible dedifferentiation into a fibroblast-like cell population. These FB-like cells remained sensitive to TGF-β, capable of re-induction into myofibroblasts [46]. MSC-secreted components, particularly MSC-derived extracellular vesicles (MSC-EVs), reduced the number of myofibroblasts and fibroblast activation protein alpha progenitors (FAPa + progenitors), without affecting their apoptosis. This reduction was possibly mediated by intercellular transfer of miR-29c and miR-129 [14].

### PPAR

PPAR, a type II nuclear receptor, plays an integral role in fatty acid storage and glucose metabolism in adipose tissue. It enhances lipid uptake and synthesis metabolism by increasing insulin sensitivity and promoting adiponectin release. The insulin-sensitizing effects of PPAR have been widely utilized in glycemic control therapies [72]. Additionally, PPAR exhibits anti-fibrotic properties. In the absence of PPAR-γ, collagen deposition, SMAD2/3 phosphorylation, and α-SMA levels increase in human and murine lung fibroblasts [48]. Research on PPAR-γ agonists, particularly pioglitazone, has revealed pathways that can inhibit or even reverse pulmonary arterial hypertension (PAH) and chronic fibroproliferative kidney diseases [72]. Long-term pioglitazone treatment can inhibit the activation of transcription factors STAT3 and EGR1 in TGFβ transgenic mouse kidneys, preventing TGFβ-induced renal fibrosis [49]. The widely used anti-diabetic drug metformin reduces α-SMA and collagen expression, alleviating bleomycin-induced pulmonary fibrosis, partly through activating PPAR signaling [73–75].

Nitrated fatty acids (NFAs) serve as unique endogenous activators of PPAR [76]. NFAs may promote myofibroblast dedifferentiation through various mechanisms, including upregulating PPAR, blocking TGF signaling and actions, converting TGF into inactive monomers, upregulating the collagen-targeting factor milk fat globule-EGF factor 8 (MFG-E8), and stimulating alveolar macrophages to uptake and degrade collagen. Experimental evidence further demonstrates that NFAs can reverse existing myofibroblast differentiation and collagen deposition in established mouse models of pulmonary fibrosis [48].

Eupatilin, a lipophilic flavonoid derived from Artemisia asiatica, acts as a PPAR-α agonist with anti-apoptotic, antioxidant, and anti-inflammatory effects [77, 78]. It directly targets pathogenic myofibroblasts stimulated by TGF-β, inducing rapid disassembly of myosin. This action leads to the disintegration of latent TGF-β complexes, thereby suppressing the induction of multiple EMT genes. Additionally, Eupatilin promotes myofibroblast dedifferentiation into intermediate cell types by blocking Smad3 phosphorylation, thus, potentially reversing fibrosis [47].

## MEK/ERK pathway

The mitogen-activated protein kinase (MAPK)/MAPK kinase (MEK)/extracellular signal-regulated kinase (ERK) signaling cascade, is closely associated with tumorigenesis, and plays a pivotal role in promoting cellular processes related to fibrosis. These processes include cell growth, proliferation, migration, prevention of apoptosis, and myofibroblast transformation [79, 80]. Inhibiting MEK has shown in suppressing lung cell proliferation induced by TGF-α, reducing the expression of matrix genes in vivo, thus preventing TGF-α-induced pulmonary fibrosis, and halting the progression of established pulmonary fibrosis [81]. Growth factors and mitogens transmit signals through this pathway, regulating gene expression in processes vital for cellular function [82]. This signaling cascade is not only implicated in tumorigenesis but also contributes to various cellular events associated with fibrosis, making it a potential therapeutic target for preventing and treating fibrotic disorders.

### FGF

Fibroblast Growth Factor (FGF) is a critical growth factor that transmits signals to the cell nucleus by binding to the FGF receptor (FGFR) and activating various signaling pathways. These pathways include Ras, MAPKs, ERKs, Src, p38 MAPKs, phospholipase-Cγ (PLCγ), Crk, jun N-terminal kinases (JNK), and protein kinase C (PKC) [83]. These pathways subsequently activate transcription factors such as ETV4 and ETV5 [84]. TGF-β1 induces the expression and release of FGF-2 in type II alveolar epithelial cells, and FGF-2 specifically counters TGF-β1-stimulated fibroblast proliferation through antibody neutralization [82]. FGFR1 signaling is also essential for fibroblast migration in IPF [85]. Elevated levels of FGF2 have been detected in bronchoalveolar lavage (BAL) from IPF patients, in lung mast cells of IPF, and in lung macrophages and mast cells of bleomycin-treated mice [86–88].Interestingly, while endogenous FGF2 is essential for the production of fibrosis, it does not participate in TGF-β1-induced fibrotic differentiation [82]. Conversely, induced FGF overexpression and exogenous FGF can counteract some pro-fibrotic effects of TGF-ß by downregulating the expression of type I collagen, α-SMA, stress fibers, and Hsp47 chaperone protein, and by increasing the expression of matrix metalloproteinases [89–91]. FGFs play a role in inhibiting TGF-β1 in various organs and cell types, involving multiple mechanisms such as ERK [92–94], focal adhesion kinase [95], Nkx2.5/Csx [96], let-7 miRNA [97], downregulation of TGFβ1 and TGFβR1 [98], and decreased Smad2 phosphorylation [99]. The intricate interplay between FGFs and TGF-β1 underscores the complexity of fibrotic processes and unveils potential avenues for therapeutic interventions.

## cAMP/PKA pathway

The cyclic AMP (cAMP)/protein kinase A (PKA) cascade is a pivotal signaling pathway. Various hormones such as prostaglandin E2 (PGE2), β2-adrenergic receptor agonists, and glucagon-like peptide-1 (GLP-1) activate adenylyl cyclase (AC) by binding to G protein-coupled receptors (GPCRs), catalyzing the conversion of ATP to cAMP [100]. cAMP then activates PKA or exchange protein activated by cAMP (Epac). PKA activation leads to the phosphorylation of its key substrates, cAMP-response element binding protein (CREB) [101, 102]. Prostaglandins, particularly prostaglandin E2 (PGE2), play a significant role in regulating fibroblast differentiation and myofibroblast dedifferentiation primarily through the activation of this pathway [89, 103, 104]. The intricate signaling events orchestrated by the cAMP/PKA cascade underscore its importance in cellular processes, providing a molecular basis for understanding the regulatory mechanisms involved in fibroblast dynamics.

### PGE

Prostaglandin E2 (PGE2) is a ubiquitous bioactive lipid mediator synthesized from arachidonic acid in the human body. It primarily activates prostaglandin receptor 2 (EP2) receptors, directly inhibiting fibroblast proliferation [105], α-SMA and collagen expression [103, 106], and myofibroblast differentiation [103, 104]. Stimulation of EP2 receptors increases intracellular cAMP production, activating protein kinase A (PKA) or exchange protein activated by cAMP (Epac) [107, 108]. In human lung fibroblasts, Epac activation and subsequent Rap1 activation account for PGE2’s anti-proliferative effects, while PKA activation and subsequent PKC-δ inhibition contribute to PGE2’s suppression of collagen expression and myofibroblast differentiation [107, 109].

Recent studies increasingly highlight PGE2’s critical role in myofibroblast dedifferentiation. Treatment of myofibroblasts with PGE2 induces dedifferentiation through the EP2/cAMP/PKA pathway, suppressing their proliferative capacity and restoring apoptosis sensitivity [89]. A bioinformatics study revealed that PGE2 can reverse the expression of 363 (62%) genes upregulated by TGF-β1 and 345 (50%) genes downregulated by TGF-β1. Genes upregulated by TGF-β1 and reversed by PGE2 are enriched in cell adhesion, contractile fibers, and myosin binding annotations, while those downregulated by TGF-β1 and reversed by PGE2 are enriched in glycoprotein, polysaccharide binding, and cell migration regulation annotations. This demonstrates significant alterations in the transcriptional program of differentiated myofibroblasts induced by PGE2 [110]. Importantly, PGE2-induced myofibroblast dedifferentiation is reversible, as dedifferentiated fibroblasts can redifferentiate into myofibroblasts upon TGF-β induction. This effect of PGE2 is associated with the inhibition of focal adhesion kinase (FAK) signaling [17].

Simultaneously, the synthetic prostaglandin E1 (PGE1) alprostadil has been shown to attenuate or reverse pulmonary fibrosis by activating prostaglandin receptors 2 and 4 (EP2 and EP4) [111].

## Other therapeutic targets and drugs

### BTZ

Bortezomib (BTZ) is a reversible inhibitor of the chymotrypsin-like activity of the 20 S core proteasome. Previous studies have indicated its ability to prevent fibrosis in the lungs, liver, and kidneys by inhibiting TGF-β1 [112–114]. Recent research has unveiled that BTZ can hinder key kinases activated by TGF-β and FGF-2, thereby suppressing fibroblast proliferation and differentiation induced by TGF-β and FGF2. BTZ also facilitates the dedifferentiation of elicited MyoFibs and IPF Fibs, rendering them more susceptible to FAS-mediated Apoptosis. At both the mRNA and protein levels, a significant decrease is observed in the expression of α-SMA and Col1a2 in MyoFibs and IPF Fibs formed after 48 h of TGF-β pretreatment. Remarkably, these anti-fibrotic effects of BTZ, both in vitro and in vivo, are unrelated to proteasome inhibition but are associated with the induction and activation of dual-specificity protein phosphatase 1 (DUSP1) [45]. It is worth noting that there is limited research on the use of BTZ for pulmonary fibrosis, and further exploration is needed to assess its safety and efficacy in this context.

### MyoD

MyoD, a myogenic regulatory factor, plays a crucial role in embryonic and adult skeletal muscle growth and differentiation [115]. Its expression is associated with the presence of myofibroblasts in tissue repair and fibrosis [116]. MyoD mediates TGF-β1-induced myofibroblast differentiation, and its endogenous downregulation regulates myofibroblast dedifferentiation and proliferation. Mitogen-induced downregulation of MyoD is mediated by the ERK1/2 MAPK signaling pathway [117]. In essence, MyoD serves as a critical switch in the differentiation and dedifferentiation of myofibroblasts, and silencing MyoD can be a therapeutic approach for treating pulmonary fibrosis.

Non-senescent lung myofibroblasts retain the ability to dedifferentiate, while aged and IPF myofibroblasts exhibit impaired dedifferentiation. In non-senescent cells, decreased levels of MyoD indicate susceptibility to myofibroblast dedifferentiation and apoptosis. In contrast, aged and IPF myofibroblasts show sustained upregulation of MyoD expression, leading to their inability to dedifferentiate and resistance to apoptosis. Genetic strategies to silence MyoD can restore susceptibility to apoptosis in IPF myofibroblasts, reinstate their ability to dedifferentiate, and partially reverse age-related persistent fibrosis in vivo [118].

### SET8

SET8, also known as PR-set7, SETD8, or KMT5A, is the sole lysine methyltransferase capable of specifically catalyzing monomethylation of lysine 20 on histone H4 (H4K20). Positioned on the nuclear chromatin, SET8 interacts not only with histone-modifying proteins but also with various non-histone factors such as p53, Twist, and Wnt. It plays a role in the regulation of crucial physiological processes like the cell cycle, DNA repair, gene transcription, and apoptosis [119]. In the context of BLM-induced lung injury, SET8 predominantly localizes to the nuclei of α-SMA-positive cells co-localized with H4K20me1. Inhibition of SET8 significantly suppresses the expression of α-SMA and ED-A-fibronectin in myofibroblasts [120].

UNC0379, identified through differentiation assays using lung myofibroblasts prepared from end-stage IPF patients, is an epigenetic modulator. By inhibiting SET8, UNC0379 markedly reduces the expression of α-SMA and ED-A-fibronectin in myofibroblasts, inducing their dedifferentiation. This effect contributes to partial alleviation of lung fibrosis without affecting the inflammatory response [120].

### Regulation of mechanical forces

The traditional perspective posits that after tissue remodeling and wound healing, myofibroblasts typically undergo apoptosis and clearance [121]. However, recent studies have gradually recognized their potential to terminate their function through phenotypic reversal, namely dedifferentiation, and this is because fibroblasts and myofibroblasts can modify their activity based on the information they receive from the mechanical environment [122]. ECM is both the result and the cause of myofibroblast differentiation. The ECM accumulated by myofibroblasts constitutes a stiff pro-fibroblast environment, promotes the continuous activation and differentiation of fibroblasts, and forms a positive feedback [123, 124]. Enhancement of mechanical stress or matrix stiffness has been shown to induce fibroblast differentiation into myofibroblasts [125, 126], and releasing mechanical stress or reducing stiffness can induce apoptosis, decrease myofibroblast contractility and α-SMA expression [127]. A research altered the ability of the cells to generate tension by altering boundary stiffness and successfully tested the hypothesis that mechanical inhibition of myofibroblast force generation leads to dedifferentiation or apoptosis depending upon the magnitude of the decrease in tension [128]. Omentin-1 can target mechanical signal accelerates fibrosis resolution and reverse established pulmonary fibrosis by promoting mechanically activated myofibroblasts dedifferentiation into lipofibroblasts, which is closely associated with ECM clearance in fibrotic tissue [129]. Paradoxically, another study suggests that pressure therapy - that is, applying appropriate mechanical pressure around the wound – can reverse the differentiation of fibroblasts into a myofibroblast phenotype and maintained fibroblasts in a quiescent state, thereby inhibiting excessive collagen deposition and scar formation [130]. Therefore, the impact of external mechanical stress and matrix stiffness on myofibroblast dedifferentiation need to be further explored, and how to change the mechanical stress and matrix hardness to achieve the cure of pulmonary fibrosis is worthy of further exploration.

## Conclusion

As the incidence and mortality rates of pulmonary fibrosis continue to rise annually, research into its pathogenesis and treatment has become increasingly urgent. Myofibroblast dedifferentiation, has emerged as a significant and promising area of study in recent years, offering potential therapeutic benefits in treating pulmonary fibrosis. Many drugs and regulatory approaches discussed in this article not only halt further progression of lung fibrosis but may even reverse established fibrosis, holding crucial implications for improving patient quality of life.

However, many specific molecular mechanisms underlying myofibroblast dedifferentiation remain unclear, particularly which mechanisms are beneficial in treating pulmonary fibrosis remain unclear. Furthermore, most current studies are still at the molecular, cellular, and animal research stages, and the bleomycin-induced lung fibrosis model used in these studies also significantly differs from human pulmonary fibrotic diseases, suggesting a lengthy road ahead for large-scale clinical trials and widespread clinical application of related drugs.

Another excellent review summarizes some effective compounds that promote myofibroblast dedifferentiation, such as amniotic membrane stromal extract, capsaicin, SNAC, GSPs and Cu/Zn SOD [131]. These compounds have shown positive effects in promoting myofibroblast dedifferentiation in other tissues and organs, and their effects in pulmonary fibrosis are awaiting further investigation.

It is anticipated that in the near future, more molecular mechanisms of myofibroblast dedifferentiation will be elucidated, and corresponding drugs will be clinically applied, aiding patients and physicians in overcoming this challenging disease.

## Data Availability

No datasets were generated or analysed during the current study.

## Abbreviations

IPF: Idiopathic Pulmonary Fibrosis
ECM: excessive extracellular matrix
TGF-β: Transforming growth factor-β
PDGF: Platelet-derived growth factor
EMT: Epithelial-mesenchymal transition
EDMT: Endothelial-mesenchymal transition
BMPs: Bone morphogenetic proteins
GDFs: Growth and differentiation factors
TβRII: TGF-β receptor II
PAI-1: Plasminogen activator inhibitor-1
CTGF: Connective tissue growth factor
AP-1: Activating protein-1
Sp1: Specificity protein 1
ERK: extracellular-signal-regulated kinases
MEK: mitogen-activated protein kinase kinase
AC: Adenylyl cyclase
PKA: Protein kinase A
PI3K: The phosphoinositide 3-kinase
Fra-2: Fos-related antigen 2
NFE2L2: Nuclear factor erythroid 2-like 2
Nrf2: Nuclear factor erythroid 2-related factor 2
ATO: Arsenic trioxide
SFN: Sulforaphane
SOCE: store-operated calcium entry
HIFi: HIF-1α inhibitors
HIF-1α: hypoxia-inducible factor-1α
BTZ: Bortezomib
PPAR: Peroxisome proliferator-activated receptor
AREs: antioxidant response elements
ER: Endoplasmic reticulum
AECs: alveolar epithelial cells
FMD: Fibroblast-to-myofibroblast differentiation
CsA: Cyclosporine A
IL-6: interleukin-6
α-SMA: α-smooth muscle actin
PAI-1: plasminogen activator inhibitor-1
NOX4: NADPH oxidase-4
ROS: reactive oxygen species
MSC: Mesenchymal stromal/stem cells
EVs: Extracellular vesicles
NFAs: Nitrated fatty acids
FGF: Fibroblast Growth Factor
PGE2: prostaglandin E2
GLP-1: glucagon-like peptide-1
GPCRs: G protein-coupled receptors
Epac: exchange protein activated by cAMP
CREB: cAMP-response element binding protein
FAK: focal adhesion kinase signaling
DUSP1: dual-specificity protein phosphatase 1
